# Baseline hemoglobin and neutrophil-to-lymphocyte ratio as prognostic biomarkers in patients with metastatic triple negative breast cancer treated with sacituzumab govitecan in second line and beyond: a real-world analysis

**DOI:** 10.1007/s10549-025-07825-0

**Published:** 2025-09-27

**Authors:** Małgorzata Pieniążek, Anna Polakiewicz-Gilowska, Manuela Las-Jankowska, Jakub Wronowicz, Michał Jarząb, Aleksandra Łacko, Mirosława Püsküllüoğlu

**Affiliations:** 1 Department of Oncology, Wroclaw Medical University, Plac Hirszfelda 12, 53-413 Wrocław, Poland; 2 Lower Silesian Comprehensive Cancer Center, 53-413 Wrocław, Poland; 3 Breast Cancer Unit, Maria Skłodowska-Curie National Research Institute of Oncology, Gliwice Branch, 44-102 Gliwice, Poland; 4 Surgical Oncology, Oncology Centre, Ludwik Rydygier Collegium Medicum in Bydgoszcz, Nicolaus Copernicus University in Torun, 85-067 Bydgoszcz, Poland; 5 Department of Clinical Oncology, Oncology Center-Prof Franciszek Lukaszczyk, 85-067 Bydgoszcz, Poland; 6 Statistical Analysis Centre, Wroclaw Medical University, 50-372 Wroclaw, Poland; 7 Department of Clinical Oncology, Kraków Branch, Maria Sklodowska-Curie National Research Institute of Oncology, 31-115 Kraków, Poland

**Keywords:** Hemoglobin, Peripheral blood markers, Sacituzumab govitecan, Metastatic triple negative breast cancer, Prognostic factor

## Abstract

**Purpose:**

This study aimed to evaluate the prognostic significance of baseline laboratory parameters and inflammatory indices in patients with metastatic triple-negative breast cancer (mTNBC) treated with sacituzumab govitecan (SG) in the second line and beyond, potentially aiding personalized patient management.

**Methods:**

This retrospective cohort study analyzed data from 83 female patients with mTNBC who initiated SG therapy at four Polish oncology centers between August 2021 and September 2024. Hematological parameters—white blood cell count (WBC), hemoglobin (Hb), platelets (Plt) and inflammatory indices—neutrophil-to-lymphocyte ratio (NLR), platelet-to-lymphocyte ratio (PLR), and systemic immune-inflammation index (SII) were assessed at baseline and before each dose of first four SG cycles. Median progression-free survival (mPFS) and overall survival (mOS) were estimated, and, as the primary objectives, associations between baseline laboratory variables and survival outcomes were assessed using multivariate Cox regression models (*α* = 0.05). Secondary objectives included assessing their associations with patient and disease characteristics, prior treatment lines, and adverse events (AEs), which were classified using the National Cancer Institute Common Terminology Criteria for AEs (NCI‐CTCAE), version 5.0.

**Results:**

The mPFS was 4.07 months (95% CI 3.05–6.18), while the mOS was 8.01 months (95% CI 6.05–9.75). Lower baseline Hb was significantly associated with shorter PFS (HR = 0.82, *p* = 0.03) but not OS. Elevated baseline NLR predicted worse OS (HR = 1.18, *p* = 0.03), while PLR and SII lacked prognostic significance. Changes in blood parameters within initial four SG cycles showed no significant correlations with survival outcomes. Furthermore, baseline hematological markers and inflammatory indices showed no significant association with clinical characteristics, prior therapy lines, tumor burden, or the occurrence and severity of AEs.

**Conclusions:**

Baseline Hb and NLR were identified as independent prognostic biomarkers in patients with mTNBC receiving SG treatment, predicting PFS and OS, respectively. Other inflammatory indices (PLR, SII) did not demonstrate prognostic relevance. Prospective validation in larger cohorts is essential to confirm these findings and potentially guide personalized treatment strategies.

**Supplementary Information:**

The online version contains supplementary material available at 10.1007/s10549-025-07825-0.

## Introduction

Triple-negative breast cancer (TNBC) is associated with the poorest prognosis among the most commonly diagnosed cancers worldwide [1]. Its inherently aggressive biology translates into unfavorable outcomes, particularly in patients with advanced disease [2, 3]. The introduction of sacituzumab govitecan (SG), an antibody–drug conjugate (ADC), into the therapeutic arsenal for metastatic TNBC (mTNBC) has significantly improved patient outcomes by prolonging progression-free survival (PFS) and overall survival (OS) [4]. However, treatment of mTNBC continues to pose a major challenge, mainly due to rapid progression and deterioration of patients’ general condition, which often limits the feasibility of subsequent therapeutic interventions. Thus, the identification of reliable biomarkers to guide therapeutic decisions, enable personalized treatment strategies, and predict responses to therapy remains an urgent clinical need**.** Given that SG has become a breakthrough option for patients with relapsed or refractory mTNBC, clarifying prognostic factors in this setting is particularly relevant. An important question is whether routinely available baseline hematologic parameters may influence treatment efficacy or the risk of toxicity, as has been demonstrated for other therapies but not for ADCs [5, 6]. To date, neither clinical trial evidence nor real-world analyses have yet clarified whether these parameters carry prognostic significance in patients receiving SG [4, 7–12].

Given that metastatic cancer is a systemic disease closely intertwined with inflammation, increasing attention has been directed toward circulating inflammatory cells and mediators that modulate the tumor microenvironment and contribute to disease progression [13, 14]. These insights have established a clear connection between tumor-induced systemic immunological alterations and peripheral blood morphological parameters, as well as their derived indices, which reflect inflammatory status and are recognized as valuable prognostic indicators [6].

Elevated white blood cell (WBC) counts generally correlate with survival outcomes, though this relationship varies significantly across their subsets [15]. Specifically, increased neutrophil counts are associated with enhanced tumor growth and metastasis, reflecting poorer clinical outcomes, whereas elevated lymphocyte counts typically predict improved survival [6, 15]. Conversely, low hemoglobin (Hb) levels, indicative of tumor-related hypoxia, facilitate tumor progression and correlate with reduced therapeutic response and worse survival outcomes [13]. Similarly, increased platelet counts promote tumor growth and are associated with adverse prognoses [6, 15, 16].

In addition to absolute cell counts, inflammatory indices such as neutrophil-to-lymphocyte ratio (NLR), platelet-to-lymphocyte ratio (PLR), and systemic immune-inflammation index (SII) have emerged as valuable prognostic markers, with elevated values consistently linked to worse clinical outcomes in metastatic disease [15, 17]. However, the majority of studies evaluating the prognostic value of inflammatory markers in breast cancer (BC) have primarily involved patients undergoing treatments with curative intent [15, 18–20]. Although increased inflammatory markers have been associated with advanced disease stages, their precise prognostic significance specifically in metastatic BC remains less explored.

The primary objective of this study was to evaluate whether baseline parameters before SG treatment—such as WBC, Hb, and Plt—as well as indices including NLR, PLR, and SII, correlate with PFS and OS. The secondary objective was to investigate the association between these initial blood parameters and selected patient- and disease-related characteristics. Additionally, the analysis included correlations with the number of prior treatment lines before SG initiation, as well as the occurrence and severity of adverse events (AEs). Finally, the dynamics of blood parameter changes during the first four cycles of SG were assessed in relation to PFS and OS.

## Materials and methods

### Study population and treatment

From August 2021 to September 2024, SG treatment was initiated in 83 female patients diagnosed with unresectable locally advanced or metastatic TNBC across four oncology centers in Poland: the Lower Silesian Comprehensive Cancer Center in Wrocław (*n* = 32), the Maria Skłodowska-Curie National Research Institute of Oncology in Gliwice (*n* = 28), the National Research Institute of Oncology in Krakow (*n* = 13) and the Oncology Center—Prof. Franciszek Łukaszczyk in Bydgoszcz (*n* = 10). During the study period in Poland, SG was reimbursed exclusively for TNBC; therefore, this real-world cohort included only TNBC patients. Patients were identified through hospital registries and met eligibility requirements consistent with the Polish drug reimbursement guidelines derived from the ASCENT trial (English translation of the inclusion and exclusion criteria for the Polish drug reimbursement program is provided in the Supplementary Appendix) [21]. Key inclusion criteria: histologically confirmed TNBC, disease lesions evaluable according to Response Evaluation Criteria in Solid Tumors (RECIST), version 1.1 [22] and Eastern Cooperative Oncology Group performance status (ECOG PS) of 0–1**,** and had previously received at least two systemic treatment lines, with at least one regimen administered in the advanced-stage setting. Treatment continued until disease progression, unacceptable toxicity, deterioration of patient condition or quality of life, patient or physician decision, or death.

The initial SG dose was typically set at 10.0 mg/kg, but physicians had discretion for dose adjustments. Dose modifications in response to AEs adhered to the guidelines outlined in the European Union Summary of Product Characteristics (SmPC) [23].

### Data collection

This retrospective cohort study was based on the data retrieved from both electronic and paper-based medical records. To ensure a structured and comprehensive analysis, the collected data were categorized into three main groups—demographic (patient age and sex), pathological (estrogen receptor (ER), progesterone receptor (PgR), human epidermal growth factor receptor 2 (HER2) status, and the Ki-67 proliferation index, assessed from either initial or most recent tumor samples) and clinical data (date of mTNBC diagnosis, sites, number and highest measure of metastatic lesions, SG therapy, AEs). Treatment-related data focused on SG administration, documenting the treatment initiation date, subsequent infusion dates, and the final dose administration. AEs were classified according to the National Cancer Institute Common Terminology Criteria for AEs (NCI-CTCAE), version 5.0 [24]. The data on grade 1 neutropenia and leukopenia were not collected, as in routine practice such events are not documented as AEs, as they do not require intervention or affect treatment decisions. Treatment efficacy was assessed using the RECIST 1.1 criteria to evaluate computed tomography or magnetic resonance images performed at least every 8 weeks in accordance with the Polish drug reimbursement guidelines. The treating physician was not permitted to extend this interval. Radiological responses were classified as complete response (CR), partial response (PR), stable disease (SD), or progressive disease (PD). The objective response rate (ORR) was defined as the proportion of patients achieving either CR or PR [22]. Additional data included details on subsequent lines of therapy following SG treatment, the date of the last clinical follow-up (FU) and the date of death, where applicable.

### Blood samples and blood counts data

Peripheral complete blood count was performed at baseline and prior to each administration SG, following standardized procedures of local laboratories and in accordance with the requirements specified in the SmPC and drug reimbursement program [21, 23]. The complete blood count included absolute values of all blood cells, which were retrospectively entered into a dedicated anonymized database. Hematological parameters were collected before each SG dose, and inflammatory indices were subsequently calculated. The NLR was calculated by dividing the neutrophil count by the lymphocyte count. PLR was calculated as the platelet count divided by the lymphocyte count. SII, a composite inflammatory index incorporating platelet, neutrophil, and lymphocyte counts, was calculated by multiplying the platelet count by neutrophil count and then dividing the result by the lymphocyte count. The dynamics of blood parameter changes were defined either as a trend or as the difference between the first measurement and the value at cycle 4. Data cut-off was October 31, 2024.

### Primary and secondary objectives

The primary objective of this study was to evaluate whether baseline hematological parameters obtained before SG treatment—such as WBC, Hb, and Plt counts—as well as inflammatory indices, including NLR, PLR, and SII, correlate with PFS and OS.

PFS was defined as the time from the initiation of SG treatment to disease progression or death from any cause, whichever occurred first. OS was defined as the time from the initiation of SG therapy until death from any cause.

The secondary objectives included evaluating the association between the baseline hematological parameters and selected patient—and disease-related characteristics. In addition, analyses encompassed correlations with the number of prior lines of therapy before SG initiation and the occurrence and severity of AEs. Finally, the dynamics of hematological parameter changes during the first four cycles of SG were assessed—defined either as trends or as differences between baseline and cycle 4 measurements—and analyzed in relation to PFS and OS.

### Statistical analysis

Continuous variables are presented as medians with interquartile ranges (IQR), and categorical variables as frequencies and percentages. Normality and homogeneity of variances were assessed using the Shapiro–Wilk and Levene’s tests, respectively. Comparisons between two groups were performed using the Wilcoxon rank-sum test or Student’s/Welch’s *t* tests depending on data distribution and variance equality. For comparisons among three or more groups, the Kruskal–Wallis, ANOVA, or Welch ANOVA tests were applied analogously. Spearman’s correlation was used to assess relationships between continuous variables selected, based on clinical relevance and prior research. Associations between explanatory variables and survival outcomes (PFS, OS) were analyzed using multivariate Cox regression models. The variables included in the multivariate Cox analysis were divided into two groups: 1.WBC, Plt, and Hb, commonly evaluated hematological parameters indicative of systemic inflammation, nutritional status, and bone marrow function; and 2. NLR, PLR, and SII**,** selected due to their recognized significance as systemic inflammatory indices reflecting immune response and tumor progression in cancer prognosis. All baseline laboratory values were analyzed as continuous variables. No a priori dichotomization or data-driven cut-offs were applied to avoid information loss and reduce bias associated with arbitrary thresholds. To prevent overfitting, we preselected predictors based on theoretical considerations and maintained a ratio of at least 10 events per predictor, adhering to the common rule of thumb for Cox regression analysis. Assumptions of proportional hazards, linearity, influential observations, and multicollinearity were verified using standard residual diagnostics (Schoenfeld, Martingale, deviance, dfbeta residuals) and Variance Inflation Factor (VIF). Analyses were performed with R (v4.4.0) and Python (v3.11.4), with a significance level of *α* = 0.05.

## Results

### Characteristics and treatment of the study population

The median age of the 83 women treated with SG was 54 years (IQR 46–65). The study cohort consisted of 67.5% postmenopausal women and 41% presented with comorbidities. Two patients initiated SG with ECOG PS 2 outside the national drug program. Only 9.6% of cases were associated with germline BReast CAncer gene (BRCA) mutation. The TNBC disease was characterized by poor prognostic factors such as grade 3 (57.8%) and the median Ki-67 proliferation index was 50 (IQR 35–72.5). Tumor burden of metastatic disease was described in details in Table 1. At the time of data collection, the mPFS was 4.07 months (95% CI 3.05–6.18), while the mOS was 8.01 months (95% CI 6.05–9.75) and the median FU time was 7.42 months (95% CI 6.07–8.96). 59 patients (71%) had completed treatment—55 (96.6%) due to disease progression, 2 (3.4%) due to death, and another 2 (3.4%) because of complications. Meanwhile, 24 patients (28.9%) remained on treatment. SG was administered across various treatment lines, with a median of 2 (IQR: 2–3). The median number of completed SG cycles was 5 (IQR 3–9) cycles. An ORR was achieved in 25 patients (30.1%), with further treatment response details provided in Table 2. The observed AE profile only partially overlapped with findings from clinical trials. The most frequently reported toxicity was neutropenia grade ≥ 2, occurring in 63.9% of cases. To manage neutropenia, 35 patients (42.2%) received granulocyte colony-stimulating factor (G-CSF) as primary prophylaxis starting on day 2 or 3 of cycle 1 due to a prior history of neutropenia or febrile neutropenia. However, another 33 patients (39.8%) required G-CSF at some point during SG treatment. A complete list of side effects is available in Table 3.

**Table 1:** Baseline patient demographics, tumor burden, and prior palliative treatments at sacituzumab govitecan initiation

Category	Parameter	*N* = 83
Patient characteristics	Median age (IQR)	54 (46–65)
	ECOG PS	*N* (%)
	0	25 (30.12)
	1	56 (67.47)
	2	2 (2.41)
	Menopausal status	*N* (%)
	Premenopausal	27 (32.53)
	Postmenopausal	56 (67.47)
	Coexisting conditions	*N* (%)
	No	49 (59.04)
	Yes	34 (40.96)
	Germline BRCA mutation carrier	*N* (%)
	No	74 (89.16)
	Yes	8 (9.64)
	Unknown	1 (1.20)
Tumor burden characteristics	Number of metastatic lesions	*N* (%)
	1–3	19 (22.89)
	4–10	37 (44.58)
	10–20	6 (7.23)
	> 20	15 (18.07)
	Only non-measurable lesions	5 (6.03)
	Unknown	1 (1.20)
	Number of metastatic sites	*N* (%)
	2	30 (36.14%)
	3	22 (26.51%)
	1	11 (13.25%)
	4	10 (12.05%)
	5	6 (7.23%)
	6	3 (3.61%)
	7	1 (1.2%)
Treatment before SG	Median number of previous palliative regimens (IQR)	2 (2–3)
	Previous chemotherapy regimens	*N* (%)
	1	41 (49.4)
	2–3	35 (42.17)
	> 3	7 (8.43)
	Previous anticancer drugs	*N* (%)
	Platins	43 (51.81)
	Taxanes	31 (37.35)
	Capecitabine	30 (36.14)
	Anthracyclines	22 (26.51)
	Gemcitabine	17 (20.48)
	Vinorelbine	11 (13.25)
	iPARP	4 (4.82)
	Pembrolizumab	4 (4.82)

*ECOG PS* Eastern Cooperative Oncology Group Performance Status, *SG* sacituzumab govitecan, *iPARP* PARP inhibitors, *N* number

Values are presented as *N* (%) unless otherwise specified. Median values are accompanied by interquartile range (IQR)

**Table 2:** Response evaluation according to RECIST criteria

Response	Number and percentage (*N* = 83, 100%)
CR	2 (2.41%)
PD	26 (31.33%)
PR	23 (27.71%)
SD	30 (36.14%)
Unknown	2 (2.41%)

*CR* complete response, *PD* progressive disease, *PR* partial response, *RECIST* response evaluation criteria in solid tumors, *SD* stable disease

**Table 3:** Reported side effects of sacituzumab govitecan treatment

Side effect	Any grade, *N* (%)	Grades and cases per side effect					
		1	2	3	4	5	Lack of data
Neutropenia	53 (63.9%)*	Not collected	16	21	16	–	–
Leukopenia	45 (54.2%)*	Not collected	32	10	3	–	–
Anemia	30 (36.1%)	5	18	7	–	–	–
Elevated liver enzymes	16 (19.3%)	3	8	4	–	–	1
Thrombocytopenia	11 (13.3%)	1	3	4	1	–	2
Diarrhea	10 (12.0%)	4	3	3	–	–	–
Nausea	9 (10.8%)	3	5	1	–	–	–
Vomiting	3 (3.6%)	–	3	–	–	–	–
Hypersensitivity reactions	2 (2.4%)	–	–	1	–	–	1
Febrile neutropenia	1 (1.2%)	–	–	–	–	1	–

*N* number

*Grade 1 events were not collected, as they are not routinely documented in clinical practice

### Study results

#### Descriptive analysis of hematologic parameters

Median values and interquartile ranges (IQR) of baseline hematologic parameters (WBC, Hb, Plt) and inflammatory indexes (NLR, PLR, SII), as well as their changes after four cycles of therapy, are presented in Table 4.

**Table 4:** Changes in hematological and inflammatory indices between baseline and cycle 4 of treatment

Parameter	Baseline, median (IQR)	Cycle 4, median (IQR)	Δ Change*, median (IQR)
WBC	5.78 (4.64–7.7)	4.62 (3.73–6.61)	– 0.47 (– 2.11–0.49)
Plt	239 (195–298)	262 (214–300)	20.5 (– 19–49.25)
Hb	11.7 (10.45–12.6)	11.05 (10.4–12)	– 0.4 (– 1.3–0.43)
NLR	3.42 (1.99–5.24)	2.39 (1.7–3.53)	– 0.61 (– 1.75–0.27)
PLR	201.94 (143.62–352.68)	257.77 (154.55–318.77)	23.22 (– 50.37–81.23)
SII	814.83 (479.52–1492.74)	575.33 (422.91–1031.35)	– 108.2 (– 404.77–183.29)

*Hb* hemoglobin, *NLR* neutrophil-to-lymphocyte ratio, *Plt* platelets, *PLR* platelet-to-lymphocyte ratio, *SII* systemic immune-inflammation index, *WBC* white blood cells

*Δ Change calculated as value at cycle 4 minus baseline

Values at cycle 4 were available for *N* = 64 patients

#### Basic hematological (Hb, WBC, Plt) parameters and survival outcomes

The Cox regression model identified Hb as a significant predictor of PFS (HR = 0.82, 95% CI 0.69–0.98, *p* = 0.03), indicating that lower Hb levels were associated with a higher risk of disease progression. In contrast, WBC and Plt showed no statistically significant association with PFS. None of the examined hematological parameters were significantly correlated with OS. All results of statistical analysis are summarized in Table 5.

**Table 5:** Multivariate Cox regression analysis for PFS and OS

Model	Outcome	Variable	Hazard ratio (95% CI)	*p* value
Model 1 (WBC + Hb + Plt)	PFS	WBC	0.98 (0.92–1.05)	0.59
		Plt	1.00 (1.00–1.00)	0.66
		Hb	0.82 (0.69–0.98)	0.03*
	OS	WBC	1.00 (0.94–1.07)	0.92
		Plt	1.00 (1.00–1.00)	0.48
		Hb	0.93 (0.77–1.12)	0.44
Model 2 (NLR + SII + PLR)	PFS	NLR	1.09 (0.93–1.27)	0.28
		PLR	1.00 (1.00–1.00)	0.97
		SII	1.00 (1.00–1.00)	0.56
	OS	NLR	1.18 (1.02–1.38)	0.03*
		PLR	1.00 (1.00–1.00)	0.70
		SII	1.00 (1.00–1.00)	0.42

*CI* confidence interval, *Hb* hemoglobin, *NLR* neutrophil-to-lymphocyte ratio, *OS* overall survival, *PFS* progression-free survival, *Plt* platelets, *PLR* platelet-to-lymphocyte ratio, *SII* systemic immune-inflammation index, *WBC* white blood cells

*p* value < 0.05 indicates statistical significance (marked with an asterisk)

#### Inflammatory indexes (NLR, PLR, SII) and survival outcomes

None of the studied inflammatory indices were significantly associated with PFS. The hazard ratios for NLR, PLR and SII were not statistically significant. NLR was found to be a significant predictor of OS (HR = 1.18, 95% CI 1.02–1.38, *p* = 0.03), suggesting that higher NLR levels were associated with a poorer prognosis. PLR and SII did not show significant associations with OS. All results of statistical analysis are summarized in Table 5.

Finally, the dynamics of blood parameter changes during the first four cycles of SG did not show significant associations with either PFS or OS.

Basic blood parameters and inflammatory indexes and selected patient- and disease-related characteristics.

No statistically significant associations were found between basic blood parameters, inflammatory indexes and age, comorbidities, Ki-67, the number of prior treatment lines, or tumor burden. However, Spearman’s correlation analysis indicated a weak negative correlation between NLR and age (*r* = − 0.13), though this was not statistically significant (*p* = 0.26). Similarly, a weak positive correlation was observed between NLR and Ki-67 (*r* = 0.1), but this too did not reach statistical significance (*p* = 0.39). In addition, NLR showed a weak positive correlation with the number of measurable lesion sites (*r* = 0.13), although again, statistical significance was not achieved (*p* = 0.23). PLR and SII exhibited similar trends.

Association between baseline blood parameters, inflammatory indexes, and the occurrence and severity of AEs.

The analysis revealed no statistically significant correlations between the baseline hematological parameters or inflammatory indices and the occurrence or severity of AEs.

No associations were found between NLR, PLR, or SII, and the frequency of, diarrhea, vomiting, nausea, hypersensitivity reactions, liver parameter abnormalities, anemia, thrombocytopenia, neutropenia, febrile neutropenia, or alopecia. Evaluation of baseline hematological parameters also showed no significant correlations with the aforementioned AEs or their severity grades. The only exception was Hb, for which ANOVA indicated a potential correlation with the occurrence of neutropenia (*p* = 0.002); however, this finding lost statistical significance after FDR adjustment (adjusted *p* = 0.15). Thus, this result should be interpreted cautiously and warrants further investigation.

## Discussion

The findings of the present study indicate that among the evaluated hematological markers, only Hb demonstrated prognostic significance for PFS, whereas no associations were observed for OS. Regarding inflammatory indices, only the NLR emerged as a potential prognostic indicator for OS, while PLR and SII did not exhibit significant prognostic value. Importantly, the dynamics of peripheral blood markers during the first four cycles of SG therapy did not show significant associations with either PFS or OS. In addition, no statistically significant relationships were identified between selected patient and disease-related characteristics. Finally, none of the analyzed peripheral blood markers showed correlation with the occurrence or severity of AEs. These findings highlight that standard hematological parameters may not be sufficient for predicting treatment-related toxicity and emphasize the necessity of identifying more sensitive biomarkers.

It should be emphasized that so far, there have been no available RWD or clinical trial results evaluating the relationship between peripheral blood inflammatory markers and treatment outcomes in patients with mTNBC receiving SG [4, 7, 8, 10–12]. Therefore, our study provides the first clinical evidence regarding the prognostic value of hematological parameters specifically in patients receiving SG therapy.

In our study, we demonstrated that lower Hb levels correlate with shorter PFS. This finding aligns with numerous studies confirming that low pre-treatment Hb levels are negatively associated with both PFS and OS across various cancers [13, 25]. Several biological mechanisms may explain why low Hb levels contribute to a shorter PFS. Anemia reduces the oxygen-carrying capacity of the blood, leading to tumor hypoxia, which plays a pivotal role in promoting tumor aggressiveness [26]. Hypoxia drives the expression of genes associated with enhanced tumor proliferation, angiogenesis, and metastatic potential, fostering a more malignant tumor phenotype [26]. Moreover, hypoxia impairs the effectiveness of cancer therapies. In chemotherapy, insufficient genation can hinder drug penetration into the tumor microenvironment and activate DNA repair mechanisms, allowing resistant cancer cell clones to survive and proliferate [27]. Furthermore, hypoxia appears to facilitate the selection of more aggressive tumor cell clones while simultaneously inhibiting apoptosis, thereby accelerating disease progression [27]. Data regarding the role of Hb in TNBC relate only to radical treatment and indicate that among patients, lower Hb levels before therapy—regardless of the treatment type—are associated with shorter survival [19, 28]. Notably, similar data are currently lacking for patients with mTNBC treated specifically with SG or other ADC therapies. Thus, our findings represent novel and clinically relevant evidence regarding prognostic factors for PFS in this specific patient population.

Interestingly, in our study lower Hb was associated with shorter PFS but not OS. This discrepancy may reflect the multifactorial nature of OS, which is influenced not only by baseline patient condition but also by subsequent treatment lines and supportive interventions administered after progression. Moreover, the relatively short follow-up and limited cohort size may have reduced the statistical power to demonstrate an association with OS. These considerations suggest that Hb may be primarily prognostic of near-term outcomes such as PFS, while its influence on long-term survival may be attenuated by other prognostic factors.

The absence of prognostic significance for WBC and Plt may result from patient-specific factors, including previous extensive treatment, heterogeneity of tumor burden, and widespread prophylactic use of G-CSF (overall, G-CSF was used in 68 (81.9%) patients), potentially affecting neutrophil and platelet dynamics [29].

Our study demonstrated also that higher pre-treatment NLR values correlate with shorter OS in the observed cohort of patients. These findings align with previous research indicating the prognostic significance of NLR in BC, spanning early-stage, locally advanced, and metastatic disease.

In early and locally advanced breast cancer, elevated NLR has been linked to more aggressive disease characteristics, including greater lymph node involvement and increased vascular invasion [20]. This association is particularly pronounced in locally advanced cases, where higher NLR levels reflect both aggressive tumor biology and a heightened inflammatory response [20]. Moreover, studies indicate that a high NLR is linked to a reduced likelihood of achieving pathological complete response (pCR) following neoadjuvant chemotherapy (NAC) [30]. In addition, lack of pCR supports the role of high NLR in chemotherapy resistance [30].

NLR has also been evaluated as a prognostic marker in metastatic and recurrent breast cancer, particularly in patients receiving chemotherapy [5]. For instance, a low NLR (< 3) has been identified as an independent predictor of improved OS in patients treated with eribulin, a finding that aligns with our results [5]. However, in the case of capecitabine, a low NLR (< 3) was associated with improved PFS, but not OS, which contrasts with our findings, where NLR had a stronger prognostic impact on OS [5]. Importantly, the prognostic significance of NLR appears to be most pronounced in ER-negative and HER2-negative BC, suggesting that its prognostic value may vary by tumor subtype [18].

In contrast, inflammatory indices such as PLR and SII did not show significant correlations with either PFS or OS. This underscores the need for careful selection of inflammatory markers in clinical practice among patients with mTNBC treated with SG. Although elevated PLR has been linked to advanced tumor stage (Stage II–IV vs. Stage I), its prognostic relevance appears stronger in hormone receptor-positive breast cancer, rather than TNBC [31]. In TNBC, elevated PLR has been linked to immune suppression, which may explain its limited prognostic influence in our study [32]. Given that ADCs represent a more targeted chemotherapy approach, the role of PLR as a predictor of response remains uncertain [6].

Similarly, the SII has shown inconsistent prognostic value across studies. Some suggest that it may be superior to both NLR and PLR in predicting prognosis [33]. However, research on SII has yielded inconsistent results [33]. Data on BC indicate that high SII correlates with shorter DFS and OS in patients receiving NAC, but there is a lack of evidence regarding its prognostic value in metastatic disease [6].

The lack of significance for PLR and SII indicates the need for cautious selection of inflammatory markers in clinical practice among patients with mTNBC treated with SG.

Conflicting and ambiguous data regarding correlations between inflammatory markers and OS, both in our study and in the existing literature, indicate the necessity for a detailed analysis of the role of increased inflammation during treatment with SG. SG is an ADC specifically targeting Trop-2, which is highly expressed in approximately 93% of TNBC cases [34, 35]. Trop-2 expression demonstrates an inverse correlation with the expression of immune system-related genes, potentially implicating its role in suppressing local immune responses within the tumor microenvironment [34]. This suggests that Trop-2 overexpression may exert immunosuppressive effects or weaken immunological activity around the tumor [34].

It can also be hypothesized that SG, through its mechanism of action, influences the expression of immune-related genes and may consequently modulate systemic inflammatory responses and inflammatory markers. However, direct correlations between blood parameters and Trop-2 expression have not yet been clearly established. The mechanism of SG action, which includes both direct cytotoxic effects on cancer cells with high Trop-2 expression and a “bystander” effect causing destruction of adjacent cancer cells with lower antigen expression, could significantly alter the tumor microenvironment [36]. This action reduces tumor heterogeneity and potentially modifies local immune responses.

Application of SG, via the mechanisms described above, may influence the expression of inflammatory markers. It is possible that the dynamics of changes in these markers during treatment, rather than baseline values alone, have greater prognostic significance. This hypothesis was not confirmed in our study, possibly due to the widespread use of granulocyte colony-stimulating factors, often administered starting from the first treatment dose.

The independence of Hb and NLR from other clinical features demonstrated in our study indicates their universal applicability as prognostic markers, primarily associated with the patient’s condition or ongoing systemic changes induced by the generalization of the neoplastic process. Their universal applicability underscores their potential clinical utility, reflecting systemic disease biology rather than specific clinical characteristics.

The absence of a significant relationship between hematological parameters and treatment-related toxicity of SG underscores the critical need to identify and validate more sensitive biomarkers that could accurately predict treatment responses and patient outcomes. The current results indicate that standard hematological indicators, such as Hb levels and NLR, though valuable prognostically, may not fully capture the complexity of biological processes involved in therapy-induced toxicity.

Future research perspectives should therefore explicitly focus on prospective validation studies aimed at confirming the prognostic utility and clinical relevance of NLR, especially in larger, multi-center cohorts. Furthermore, comprehensive analyses exploring the molecular and pathophysiological mechanisms underlying the association between Hb levels and disease progression in SG-treated patients are warranted. Such research could clarify whether Hb actively participates in tumor progression pathways or merely reflects general patient condition and tumor burden.

Considering their practical advantages, including low cost, widespread availability, and ease of implementation, Hb and NLR represent highly attractive, clinically applicable markers for risk stratification and prognostic assessment in routine practice for mTNBC patients treated with SG. However, advancing their clinical application will require rigorous validation through well-designed prospective trials. Additionally, integration of Hb and NLR with novel molecular and genetic markers might significantly enhance their predictive accuracy, ultimately leading to improved, individualized patient management strategies in clinical oncology practice.

### Study limitations

Several limitations of our study should be explicitly acknowledged. First, the retrospective nature of the analysis inherently carries a risk of selection bias, limiting the strength of causal inferences and necessitating caution when generalizing the findings. The relatively small sample size further restricts the statistical power, potentially obscuring subtle yet clinically relevant associations. Moreover, the heterogeneity within the patient cohort—in terms of previous treatments, baseline clinical characteristics, and variations in tumor burden—could introduce additional confounding factors, influencing both prognostic outcomes and biomarker values. The widespread use of G-CSF in the analyzed patient group might have affected peripheral blood counts, especially neutrophil levels, thereby limiting the interpretability of inflammatory indices such as NLR, PLR, and SII.

In addition, variability in laboratory methodologies across different institutions could have contributed to discrepancies in hematological parameter assessments, introducing measurement bias. Radiological evaluations according to RECIST criteria were performed by multiple radiologists without centralized review, potentially leading to inter-observer variability in assessments of PFS. Such variability could impact the accuracy and consistency of the reported outcomes. Furthermore, the relatively short follow-up period should be acknowledged, as 28.9% of patients were still receiving SG therapy at the time of data cutoff, which may limit the robustness of PFS estimates. The retrospective design also complicates the assessment of adverse events, which may have been underreported or inconsistently documented in medical records. This could explain the differences observed in AE distribution compared with the ASCENT study data. Lastly, due to the lack of longitudinal and standardized assessments of inflammatory and hematological parameters across the entire treatment course, our study may have missed capturing clinically relevant temporal dynamics in these biomarkers.

Therefore, prospective studies with standardized protocols, centralized radiological assessments, longer follow-up, and larger, more homogeneous cohorts are warranted to validate our findings and refine the clinical applicability of hematological and inflammatory biomarkers in patients with mTNBC undergoing SG therapy.

## Conclusions

Overall, our findings suggest that lower Hb levels were associated with shorter PFS, while higher NLR values were associated with shorter OS. These results support a potential prognostic significance of Hb and NLR in mTNBC patients prior to starting SG treatment. However, given the retrospective nature of our analysis, the clinical relevance of these inflammatory indices requires cautious interpretation and further investigation, particularly regarding their prognostic value in response to ADC therapy. Additional prospective research involving larger cohorts is warranted to confirm these preliminary associations and better understand the underlying biological mechanisms.

## Supplementary Information

Below is the link to the electronic supplementary material.

Supplementary file1 (DOCX 20 KB)

## Data Availability

The data generated in the present study are included in the text and tables of this article. Interested parties can obtain of statistical analysis details access to the data by contacting the corresponding author upon request.
