# Supplementary material for: Baseline hemoglobin and neutrophil-to-lymphocyte ratio as prognostic biomarkers in patients with metastatic triple negative breast cancer treated with sacituzumab govitecan in second line and beyond: a real-world analysis
Source: Breast Cancer Res Treat. 2025 Sep 27;214(3):397–407. doi: 10.1007/s10549-025-07825-0 (PMC12583401; doi:10.1007/s10549-025-07825-0)
Supplement: Supplementary file 1 — Supplementary file1 (DOCX 20 KB) [file 10549_2025_7825_MOESM1_ESM.docx]

Supplementary Appendix 1

**Methods**

Inclusion and exclusion criteria in Polish drug reimbursment program

Patients eligible for enrollment must fulfill all the criteria listed below:

1. Adult patients aged 18 years or older.
2. Histologically confirmed diagnosis of advanced breast cancer, classified as either: a) metastatic breast cancer (stage IV), or b) locally advanced breast cancer (stage III), for which radical local interventions (surgery or radiotherapy) are ineffective or unfeasible.
3. Histologically confirmed diagnosis of triple-negative breast cancer (TNBC).
4. Documented absence of steroid hormone receptor expression (estrogen and progesterone receptors below 1%).
5. Documented absence of HER2 overexpression (immunohistochemistry score of 0 or 1+) or HER2 gene amplification (negative ISH result).
6. Presence of **disease lesions evaluable** according to RECIST 1.1 criteria.
7. ECOG performance status ranging from 0 to 1.
8. Neither pregnant nor breastfeeding.
9. No life-threatening visceral metastases.
10. Lack of significant comorbidities contraindicating treatment, as assessed by the treating physician based on the applicable Summary of Product Characteristics (SmPC) and national oncology guidelines.
11. No known contraindications to the administered drug.
12. Absence of untreated or progressive CNS metastases, significant neurological symptoms, or requirement to increase corticosteroid dosage within one month before the initiation of treatment.
13. Adequate organ function, confirmed through laboratory tests, enabling safe therapy initiation according to the treating physician’s judgment.
14. For patients with concurrent malignancies or a history of other cancers, eligibility requires approval from the National or Regional Consultant, contingent upon previous treatment being radical or achieving complete remission.

All of these criteria must be concurrently satisfied. Patients who previously received treatment through alternative funding sources may also join the program, provided they met the above conditions at the time their treatment began.

Exclusion Criteria: Patients will be excluded from the program in cases of:

1. Confirmed disease progression.
2. Clinically significant decline in patient health status due to cancer, even if progression is not radiologically or clinically evident.
3. Life-threatening treatment-related toxicity according to the current CTCAE criteria.
4. Recurrent or intolerable grade 3–4 adverse events related to treatment (treatment may resume if symptoms resolve or reduce to grades 1–2).
5. Decline in performance status to ECOG 3–4.
6. Documented hypersensitivity to the medication, murine protein, or any of its excipients, prohibiting ongoing treatment.
7. Clinically significant reduction in the quality of life, as judged by the treating physician.
8. Pregnancy or breastfeeding, unless continuation of treatment is justified by a risk-benefit assessment conducted by both the treating physician and the National or Regional Consultant.
